# Outcomes in Severe COVID-19 Patients Following Percutaneous Versus Open Surgical Tracheostomy: An Analysis of Clinical and Prognostic Indicators

**DOI:** 10.7759/cureus.32487

**Published:** 2022-12-13

**Authors:** Ali Akbari, Ramin Shekouhi, Vahid Khaloo, Tahereh Shamsi, Maryam Sohooli, Seyed Vahid Hosseini, Leila Ghahramani

**Affiliations:** 1 Department of Surgery, Shiraz University of Medical Sciences, Shiraz, IRN; 2 Colorectal Research Center, Department of Surgery, Shiraz University of Medical Sciences, Shiraz, IRN; 3 Department of Anesthesiology, Shiraz University of Medical Sciences, Shiraz, IRN; 4 Student Research Committee, Shiraz University of Medical Sciences, Shiraz, IRN

**Keywords:** mechanical ventilation, percutaneous tracheostomy, open tracheostomy, tracheostomy, covid-19

## Abstract

Background and objective

Tracheostomy in coronavirus disease 2019 (COVID-19) patients can be performed in cases of prolonged intubation or in patients with a known difficult airway. Tracheostomy is usually performed by two main approaches: open surgery or percutaneous endoscopic insertion. However, few studies have compared these two techniques in severe COVID-19 patients. The objective of the present study was to compare the efficacy of the two main methods of tracheostomy among patients with severe COVID-19 infection. We also aimed to investigate the impact of various lab data and medications on patient outcomes.

Materials and methods

We included all symptomatic severe COVID-19 patients in need of prolonged mechanical ventilation. We examined the patients' past medical history, arterial blood gas (ABG) analysis, laboratory workups, and medication history. We calculated the PaO_2_/FiO_2_ ratio as an index to evaluate the severity of acute respiratory distress syndrome (ARDS).

Results

During the study period, 72 patients with severe COVID-19 underwent tracheostomy tube insertion. The average age of participants was 58.93 ±15.27 years; 44 (61.1%) were male and 28 (38.9%) were female. Of note, 54 (75.0%) patients passed away and only 18 (25.0%) survived. Among the survivors, 13 (29.5%) were men and five (17.9%) were women. The study showed a significantly higher mortality rate (23, 92.0%) among patients who underwent open surgery compared to those who received percutaneous surgery (31, 65.9%) (p=0.01).

Conclusion

Based on our findings, percutaneous endoscopic tracheostomy seems to be the superior approach compared to open tracheostomy. Other predictive factors associated with patient outcomes included levels of HCO_3_, FiO_2_, PaCO_2_, and PaO_2_/FiO_2_ ratio.

## Introduction

Coronavirus disease 2019 (COVID-19), caused by severe acute respiratory syndrome coronavirus 2 (SARS-CoV-2), was first reported in late 2019 in Wuhan, China, and has led to one of the largest known pandemics worldwide. According to the World Health Organization (WHO), as of April 14, 2022, there have been over 500 million confirmed cases of COVID-19, including more than six million deaths globally [1]. Moreover, the pandemic has put an unprecedented burden on hospitals and ICUs. COVID-19 is associated with a wide spectrum of clinical manifestations, ranging from being asymptomatic to severe forms such as severe acute respiratory failure, septic shock, kidney failure, pneumonia, multiple organ dysfunction, and even death [2]. According to WHO, about 14% of patients manifest a severe disease. Infected patients may need respiratory support based on their disease severity, including supplemental oxygen therapy, continuous positive airway pressure (CPAP), and invasive mechanical ventilation and intubation. Hence, some of these patients who need prolonged intubation might benefit from tracheostomy [3].

Tracheostomy in COVID-19 patients can be performed in cases of prolonged intubation for more than 21 days and in patients with a known difficult airway [4]. According to COVID-19 guidelines, it is better not to perform a tracheostomy before 14 days of mechanical intubation due to the high risk of viral transmission. Tracheostomy may be performed even before 21 days in cases that require deep sedation and pulmonary hygiene. Tracheostomy has several proven benefits to patients, such as lower rates of laryngeal injury, enhanced pulmonary hygiene, decreased need for deep sedation, and shortened duration of ventilation and length of stay in ICUs [5]. Furthermore, it can provide additional benefits including easier airway suctioning, decreased occurrence of tracheal stenosis, and avoiding pressure-induced trauma (both to the trachea and oral cavity). However, it comes with some caveats such as the increased chance of bleeding, tracheal laceration, oesophageal injury, pneumothorax, pneumomediastinum, long-term laryngotracheal stenosis, fistula formation, and vocal cord paralysis [6]. Conventionally, open tracheostomy was generally preferred for patients in need of prolonged mechanical ventilation, mainly due to better visibility during the procedure and greater bleeding control. However, percutaneous tracheostomy has recently gained popularity due to the fact that the procedure can be performed at the bedside, lower rates of surgical site infection, and its minimally invasive nature. However, this method is also associated with a few limitations that can make this surgical modality difficult to perform, such as neck anatomical variations, obesity, and previous history of tracheostomy [7].

Multiorgan failure (lung, heart, and kidney failure) in severe COVID-19 may result in electrolyte and acid-base disorders. Most of these acid-base disorders are mild and asymptomatic. However, PH and bicarbonate (HCO_3_) alteration in critically ill patients can be associated with adverse outcomes [8]. Based on recent studies, respiratory and metabolic alkalosis are the most common acid-base disorders and lead to a higher mortality rate. Therefore, arterial blood gas (ABG) and various laboratory parameters can help in the monitoring, disease staging, and prognostication of COVID-19 patients [9]. SARS-CoV-2 infection can also lead to endothelial dysfunction, microvascular inflammation, and platelet aggregation. These changes activate the coagulation cascade and hence may cause coagulopathy [10]. COVID-19-associated coagulopathy (CAC) causes venous and arterial thrombolytic events and is one of the main poor prognostic factors. For this reason, prophylactic and therapeutic anticoagulation therapy is recommended [11]. The objective of the present study is to compare the effectiveness of the two main methods of tracheostomy (open vs. percutaneous) among patients with severe COVID-19 infection. We also aimed to investigate the impact of various lab data and medications on patient outcomes.

## Materials and methods

Study design

We employed a cross-sectional design for this study. The Ethical Committee of Shiraz University of Medical Sciences provided the ethical approval for the study (approval no: IR.SUMS.REC.1399.1071). The study was conducted from January 10, 2020, to September 13, 2021, to determine factors associated with mortality among COVID-19 patients who underwent tracheostomy insertion. This study has been reported in line with the STROCSS criteria [12].

Selection of participants

We included patients treated in the ICUs of the two main COVID-19 referral centers at our institution. All symptomatic severe COVID-19 patients with the need for prolonged mechanical ventilation were included. Severe COVID-19 infection was defined in our study as follows: (1) SpO_2_ below 94% on room air at sea level, (2) respiratory rate above 30 breaths/minute, (3) ratio of arterial partial pressure of oxygen to fraction of inspired oxygen (PaO_2_/FiO_2_) below 300 mmHg, or (4) lung infiltration above 50%. The infection in all patients was confirmed via polymerase chain reaction (PCR). We included all ICU-admitted adult COVID-19 patients (age ≥18 years) who were either intubated or on alternative ventilatory support. We excluded patients aged less than 18 years of age, and those who did not give consent for enrolment. Finally, based on a two-sided type-I error at 0.05, a total sample size of 72 patients was calculated.

Data collection

The patients were then evaluated by a surgical and an ICU specialist blinded to the patients' paraclinical data and medication histories. They obtained demographics and medical comorbidities from both the Research Patient Data Repository (RPDR) and through manual chart review. We determined the patients' past medical histories, ABG analysis, laboratory workups, and medication history. We calculated the PaO_2_/FiO_2_ ratio as an index to evaluate the severity of acute respiratory distress syndrome (ARDS) with a cut-off of 300. We measured both the in-/out-hospital mortality of patients. The two methods of tracheostomy insertion were performed by the same surgical staff, to decrease the probability of confounding bias. The surgical techniques for tracheostomy insertion were open surgical and percutaneous endoscopic tracheostomy. Also, the timing for the tracheostomy procedure was categorized into early (≤14 days after intubation) and late (≥14 days after intubation). The bedside percutaneous approach was the preferred method for tracheostomy insertion. However, patients with neck anatomical variations, cervical instability, and infection at the insertion site underwent an open surgical approach.

Data analysis

We used SPSS Statistics software version 25 (IBM Corp., Armonk, NY) for descriptive analysis of patients’ baseline demographics and other variables. The data were analyzed based on frequency, frequency percentage, and cumulative frequency percentage. Quantitative variables were reported as mean ±SD and qualitative variables were reported as numerical (percentage) data. We used the independent t and chi-square tests for the evaluation of any possible association between quantitative variables and patient outcomes. Also, the paired t-test was used for the assessment of changes in laboratory workups following tracheostomy. Additionally, the Spearman coefficient was used to evaluate the relationship between the variables. A p-value lower than 0.05 was considered statistically significant.

## Results

Patient demographics and outcomes

Patients who were admitted from January 10, 2020, to September 13, 2021, were included in this cross-sectional study. During the study period, 72 patients infected with severe COVID-19 underwent tracheostomy tube insertion. Additionally, 68 (94.4%) patients were intubated before tracheostomy insertion. The average age of participants was 58.93 ±15.27 years; 44 (61.1%) were male and 28 (38.9%) were female. The mean hospital stay was 39.2 ±35.58 days (range: 3-264 days). Also, the interval between intubation and tracheostomy ranged from 1 to 62 days, with a mean of 16.91 ±10.56 days. Also, our results showed that 32 (44.4%) patients underwent early tracheostomy (≤14 days after intubation) insertion. Baseline patient demographics are shown in Table 1. Initially, 49 patients were diagnosed via PCR test, while the rest were diagnosed via high-resolution CT (HRCT) without the PCR test.

**Table 1:** Baseline patient demographic information SD: standard deviation; T: tracheostomy

Variables	Total, n (%)	Age, years, mean ±SD	Hospital stay, days, mean ±SD	Early T (≤14 days), n (%)	Late T (>14 days), n (%)	Mortality, n (%)
Sample size	72 (100%)	58.93 ±15.27	39.2 ±35.58	32 (44.4%)	40 (55.6%)	54 (75.0%)
Gender						
Male	44 (61.1%)	61.1 ±15.7	43 ±43.1	18 (40.9%)	26 (59.1%)	31 (70.4%)
Female	28 (38.9%)	55.5 ±13.9	32.8 ±13.9	14 (50.0%)	14 (50.0%)	23 (82.1%)

Unfortunately, 54 (75.0%) of patients passed away and only 18 (25.0%) survived. Among the survivors, 13 (29.5%) were men and five (17.9%) were women. Nine (28.1%) patients who underwent early tracheostomy insertion and nine (25.0%) with late tracheostomy insertion survived. There was no statistically significant association between the time of tracheostomy and survival (p=0.77). Also, there was no difference in the survival of patients with very early (≤7 days) compared with early/late tracheostomy insertion (p=0.92). The most common patient-specific comorbidity was hypertension (33.6%), followed by diabetes mellitus (28%), ischemic heart disease (18.2%), and dyslipidemia (15.4%).

Moreover, there was no statistically significant association between patient age and outcomes (p=0.78). Also, our results showed that there was no significant association between the final outcome and the sex of patients (p=0.242), and there was no association between the duration of hospital stay and patient outcomes (p=0.688).

Open vs. percutaneous tracheostomy insertion

As shown in Table 2, 28 (59.6%) male and 19 (40.4%) female patients underwent endoscopic percutaneous tracheostomy and the remaining patients underwent open tracheostomy insertion (Table 2). The mean age of patients with the open procedure was 61.5 ±17.48 years and that of patients who received percutaneous surgical tracheostomy was 57.5 ±13 years. Our results demonstrated that there was no association in terms of age or gender with regard to the type of surgical interventions. The mean hospital stay duration after tracheostomy insertion was 30.18 ±12.2 and 44.4 ±43.6 days in open surgery and percutaneous method, respectively. Accordingly, there were no significant differences between the two groups in terms of the duration of hospital admission (p=0.41). However, our study showed a significantly higher mortality rate (23, 92.0%) among patients undergoing open surgery compared with those undergoing percutaneous surgery (31, 65.9%) (p=0.01).

**Table 2:** Patient characteristics based on the tracheostomy approach SD: standard deviation

Variables	Open surgical tracheostomy (n=25)	Endoscopic percutaneous tracheotomy (n=47)	P-value
Gender			
Male, n (%)	16 (64.0%)	28 (59.6%)	0.71
Female, n (%)	9 (36.0%)	19 (40.4%)	0.82
Age, years, mean ±SD	61.5 ±17.48	57.5 ±13.86	0.37
Hospital stay, days, mean ±SD	30.18 ±12.2	44.4 ±43.6	0.41
Mortality, n (%)	23 (92.0%)	31 (65.9%)	0.01
Duration of Intubation before tracheostomy, days, mean ±SD	21.8 ±11.7	14.7 ±9.30	0.43

Patients' laboratory data

As illustrated in Table 3, there was a significant association between ABG analysis findings and final outcomes in survived patients. Our results showed that PaCO_2_ levels changed from 46.29 ±14.60 before tracheostomy insertion to 43.40 ±12.58 after the insertion, which was statistically significant (p=0.033). Also, the HCO_3_ level changed from 15.8 to 48.4 after tracheostomy insertion (p=0.039). All of these significant indicators were lower in patients who remained alive. Accordingly, there was a significant association between changes in PaCO_2_ before and after tracheostomy insertion with a p-value of 0.039. However, there was no significant association regarding other lab data as demonstrated in Table 3.

**Table 3:** Descriptive analysis of laboratory variables and their association with patient outcomes *P-value was assessed using independent t-test and paired t-test T: tracheostomy

Laboratory variables	Mean		P-value*	Mean before T			Mean after T		
	Before T	After T		Alive	Dead	P-value*	Alive	Dead	P-value*
Gas analysis									
RR	18	18.6	0.86	18	18.08	0.96	17	18.87	0.35
PH	7.4	7.3	0.8	7.42	7.39	0.24	7.4	7.39	0.622
FiO_2_	71.27%	74.00%	0.28	59.17%	75.38%	0.01	60.36%	77.75%	0.01
HCO_3_	28.4	26.9	0.13	25.6	29.4	0.03	23.7	27.92	0.088
PaCO_2_	46.2	43.2	0.03	40.5	48.2	0.04	37.6	45.04	0.033
TV	439.6	440.2	0.63	441	439.2	0.92	437.5	440.56	0.916
PEEP	7.23	7.22	0.42	5.5	7.7	0.004	5.92	7.55	0.013
PaO_2_	66.1	64.9	0.69	69.3	65.1	0.56	68.7	63.7	0.548
PaO_2_/FiO_2_	112.65	96.19	0.033	147.59	101	0.43	125.84	86.3	0.49
Blood analysis									
BUN	29.5	30.9	0.25	27.3	30.3	0.53	29.56	31.4	0.736
Cr	1.2	1.2	0.22	1.1	1.2	0.85	1.394	1.256	0.658
Na	138.3	138.9	0.16	136.4	138.9	0.17	137.26	139.533	0.169
K	4.1	3.9	0.29	4.04	4.1	0.86	4.156	3.91	0.182
Ca	8.1	8.1	0.84	7.8	8.1	0.13	8.11	8.07	0.861
Ph	3.4	3.2	0.34	3.02	3.5	0.27	3.23	3.22	0.973
LDH	997.9	1001.3	0.55	1319.6	890.7	0.13	1101.9	932.77	0.499
D-dimer	N/A	3116.37	N/A	N/A	N/A	N/A	5384.7	2188.42	0.48
Fibrinogen	N/A	446.7	N/A	N/A	N/A	N/A	468.87	438.52	0.4

**Figure 1:**
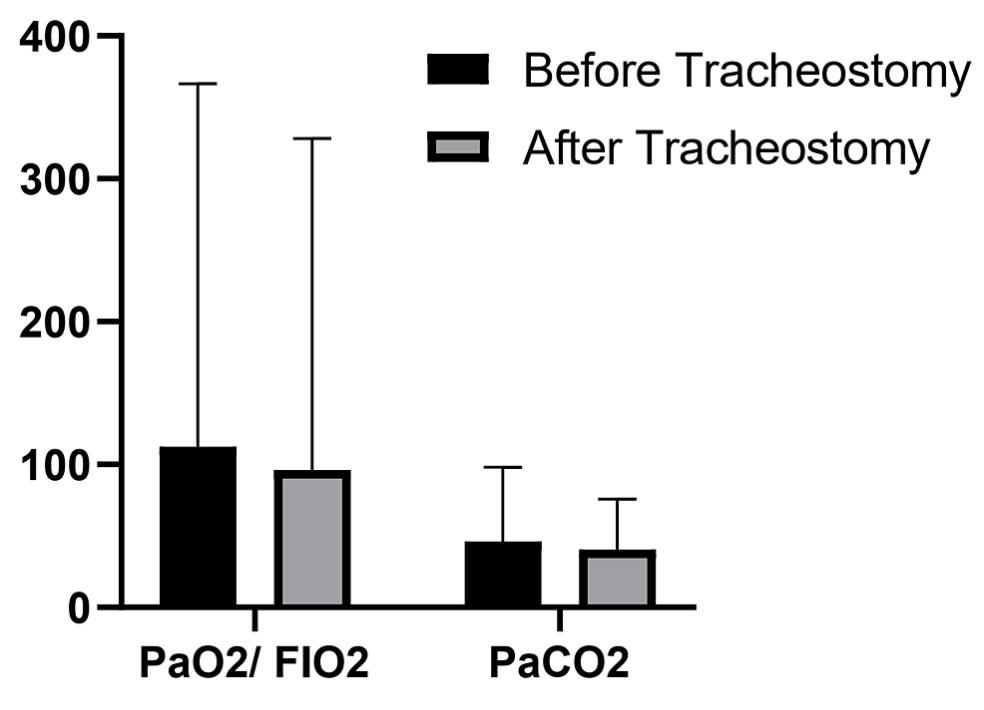
Comparison between PaCO2 and PaO2/FiO2 ratio before and after tracheostomy

**Figure 2:**
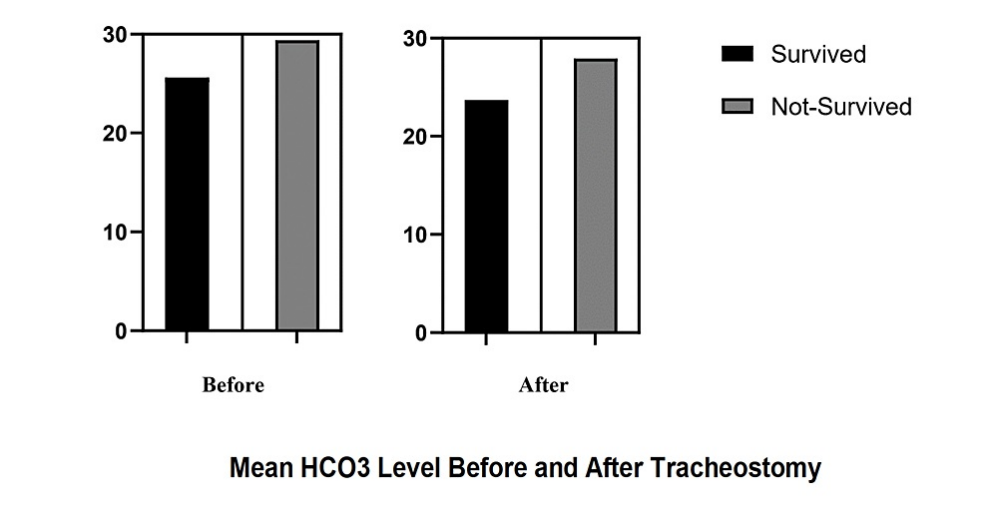
Mean HCO3 levels before and after tracheostomy in survived and non-survived patients

**Figure 3:**
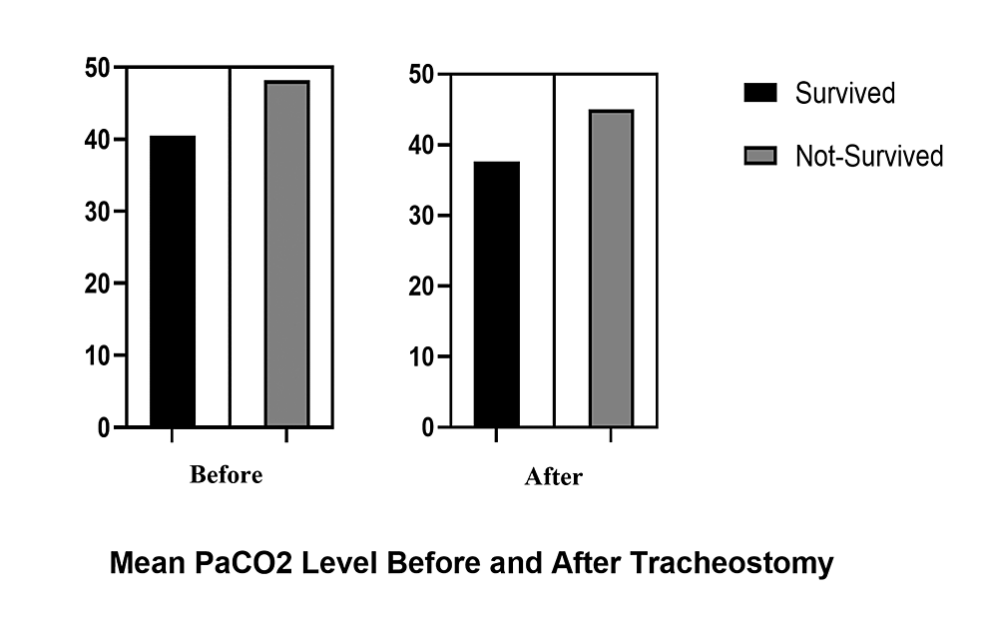
Mean PaCO2 levels before and after tracheostomy in survived and non-survived patients

Figure 1 below shows the comparison between PaCO_2_ and PaO_2_/FiO_2_ ratio before and after tracheostomy. Figure 2 shows the mean HCO_3_ levels before and after tracheostomy in survived and non-survived patients, whereas Figure 3 depicts the mean PaCO_2_ levels before and after tracheostomy in survived and non-survived patients.

Assessment of the association between patients' medications and final outcomes

The association between medications and patients’ final outcomes is presented in Table 4. Mostly, patients received medications including vancomycin (30.5%), meropenem (29.1%), midazolam (25%), enoxaparin sodium (23.6%), linezolid (22.2%), levofloxacin (22.2%), furosemide (20.8%), acetazolamide (19.4%), prednisolone (18%), morphine (16.6%), and heparin (15.2%). Patients receiving heparin, linezolid, and meropenem had a better chance of surviving (p=0.001, 0.037, and 0.018, respectively) (Table 4). There was no significant association between survival and the other medications. Also, it is worth mentioning that consuming midazolam was inversely related to survival (p=0.001).

In this study, 59 (81.9%) of acutely ill patients were prescribed antibiotics due to suspected or documented secondary infections. The most common antibiotic was carbapenems (27, 37.5%), followed by vancomycin (22, 30.55%), linezolid (16, 22.22%), and levofloxacin (16. 22.2%).

**Table 4:** Frequency of common medications and its association with patient outcomes *Chi-square test was used for evaluating any possible statistically significant association. P-value <0.05 was considered statistically significant

Medication	Total, n (%)	Outcomes, n (%)		P-value*
		Alive	Dead	
Anticoagulation	31 (43.05%)	13 (41.9%)	18 (58.1%)	0.004
Enoxaparin sodium	17 (23.61%)	4 (23.5%)	13 (76.5%)	0.435
Heparin (UFH)	11 (15.27%)	7 (63.6%)	4 (36.4%)	0.001
Rivaroxaban	3 (4.1%)	2 (66.6%)	1 (33.3%)	0.08
Corticosteroids	21 (29.2%)	6 (28.6%)	15 (71.4%)	0.61
Prednisolone	13 (18.05%)	6 (8.33%)	7 (9.72%)	0.081
Hydrocortisone	6 (8.3%)	0 (00.0%)	6 (100.0%)	0.14
Dexamethasone	2 (2.8%)	0 (00.0%)	2 (100.0%)	0.41
Antibiotics:	59 (81.9%)	17 (28.8%)	42 (71.2%)	0.11
Carbapenem	27 (37.5%)	11 (40.7%)	16 (59.3%)	0.017
Vancomycin	22 (30.55%)	5 (22.7%)	17 (77.3%)	0.382
Linezolid	16 (22.22%)	7 (43.7%)	9 (56.3%)	0.037
Levofloxacin	16 (22.2%)	2 (12.5%)	14 (87.5%)	0.19
Others				
Acetazolamide	14 (19.44%)	4 (28.6%)	10 (71.4%)	0.370
Furosemide	15 (20.83%)	2 (13.3%)	13 (86.7%)	0.082
Morphine	12 (16.66%)	2 (16.6%)	10 (83.4%)	0.206
Midazolam	18 (25%)	1 (5.5%)	17 (94.5%)	0.001

## Discussion

This cross-sectional study aimed to evaluate predictive factors related to outcomes (short- and long-term) in patients with severe COVID-19 infection who underwent tracheostomy tube insertion. This is a unique study of its kind to describe the effectiveness of frequently used medications in the treatment of severe COVID-19. The major indication of tracheostomy insertion remains the need for prolonged mechanical ventilation in patients. Although tracheostomy is associated with minimizing translaryngeal endotracheal tube complications (such as upper airway obstruction), more than 50% of COVID-19 patients needing tracheostomy died during hospitalization. During our study period, the majority of tracheostomy patients passed away, which corresponded to an exceedingly high mortality rate of 75%. Our mortality rate is much higher than that documented in the literature in the west. Ahmed et al. [13] reported a mortality rate of 33% in COVID-19 patients with tracheostomy in the United States. A small survey in India demonstrated a mortality rate of 46.1-53.8% among COVID-19 patients with tracheostomy [14].

In terms of the timing of tracheostomy, early and late tracheostomy were suggested for patients with prolonged intubation. For instance, Miles et al. [15] recommended late tracheostomy beyond 21 days of intubation for severe cases to decrease the chances of infection transmission to healthcare workers. Other studies favored tracheostomy insertion at an earlier time frame of 10-14 days [16]. However, in our study, we observed no difference in terms of survival between early (≤14 days) and late (>14 days) tracheostomies. Also, there was no difference in the survival of patients with very early (≤7 days) compared with early/late tracheostomy insertion. This finding could be attributed to the fact that in the early stages of COVID-19 infection, the primary disease is still evolving. Thus, the overall survival of patients cannot be attributed to the timing of tracheostomy per se.

Another factor that can be associated with the overall survival of COVID-19 patients is the type of surgical procedure for tracheostomy insertion. Our study demonstrated that open tracheostomy was associated with a significantly higher mortality rate with a survival rate of only 8%. However, our results contrast with the existing literature. A study by Botti et al. [17] showed that there are no associations between the type of surgical modality for tracheostomy insertion and postoperative complications. The authors concluded that the selection of surgical technique is mainly based on patient-specific conditions including neck anatomical variations (ease of finding landmarks), obesity, and the availability of experienced surgeons. Also, a meta-analysis by Klotz et al. [18] compared percutaneous tracheostomy with the open surgical method. They demonstrated that although open surgery takes longer to perform, there are no significant differences in major postoperative complications between these tracheostomy techniques. The high incidence of mortality observed in the open surgical group in our study could be attributed to the fact that most patients in this group had a higher rate of comorbidities and were not eligible to undergo percutaneous insertion. Thus, a possible selection bias should be taken into consideration.

As the severity of COVID-19 progresses, the need for prolonged intubation and ICU admissions increases as well. Acid-base disturbances are one of the most common findings in ICU patients. Very few studies have evaluated the ABG analysis of ICU-admitted COVID-19 patients. Previous studies have observed higher PH and HCO_3_ levels among COVID-19 survivors [19]. The main reason behind this alkalemia seems to be mineralocorticoid excess caused by overactive renin-angiotensin system (RAS) activation. However, our study demonstrated no significant difference in the PH levels of patients before and after tracheostomy insertion. Also, no difference was observed between survivors and non-survivors in terms of PH levels. However, HCO_3_ levels were significantly higher in non-survivors than survivors, which contrasts with previous studies [20]. Interestingly, our study revealed that blood HCO_3_ levels decreased following tracheostomy. Although statistically insignificant, this finding suggests that tracheostomy in patients with severe COVID-19 infection might lead to RAS downregulation and improved outcomes.

Interstitial involvement, V/Q mismatch, and intrapulmonary shunting are the main causes of hypoxemia and decreased PaO_2_ among severe COVID-19 patients. In our study, we assessed different ABG variables and their potential impact on patient survival. Our available data showed that FiO_2_ effectively predicted mortality among severe COVID-19 patients. Regardless of tracheostomy, all non-survivors required higher FiO_2_ levels to maintain hemostasis (p=0.01). However, it seems that tracheostomy has an insignificant impact on FiO_2_ levels. The PaO_2_/FiO_2_ ratio is a commonly used index to evaluate the severity of ARDS [21]. Previous studies have suggested that a PaO_2_/FiO_2_ ratio of less than 300 strongly contributed to poorer outcomes [22]. However, due to severe V/Q mismatch and intrapulmonary shunting caused by COVID-19 involvement, a few studies have suggested nonlinear relations between PaO_2_ and FiO_2_ [23]. Our study demonstrated that long-term mechanical ventilation with a tracheostomy is associated with lower FiO_2_ levels. In line with previous studies, we observed that FiO_2_ levels are far lower in non-survivors compared to survivors.

Regarding PaCO_2_ values, our study showed that PaCO_2_ decreases insignificantly following tracheostomy, which is mainly due to an increase in respiratory rate as shown in Table 3. Contrary to previous hypotheses, our study showed that hypercapnia (regardless of tracheostomy) was more frequently observed among non-survivors. Thus, our study showed that higher levels of HCO_3_, FiO_2_, and PaCO_2_ and a lower PaO_2_/FiO_2_ ratio are the main predictors of adverse outcomes.

Unlike the aforementioned significant findings on ABG analysis, blood workups showed no association with survival in this study. Interestingly, almost all patients (69, 95.8%) enrolled in our study showed higher-than-normal D-dimer levels (normal range: 250-500 ng/mL). Similar findings were observed in terms of lactate levels. These findings were in line with the previous studies. In fact, Helms et al. [24] concluded that elevated D-dimer and fibrinogen levels were observed in more than 95% of COVID-19 patients. Coagulation and thrombotic disorders are quite common and have been noted in various studies [25]. Current evidence regarding the indications of prophylactic anticoagulation therapies and the optimal dosage varies widely in the literature. However, most studies in the literature suggest that prophylactic anticoagulation pharmacotherapies could be considered for all hospitalized ICU-admitted COVID-19 patients unless a contraindication existed [26]. During our study period, 31 (43.05%) cases were receiving anticoagulation therapy, with enoxaparin sodium being the most common medication used. It is noteworthy that the rest of the participants were not eligible for starting an anticoagulative medication. In line with previous studies, we found that anticoagulation therapies (either LMWH or UFH) are associated with better outcomes and lower in-hospital mortality rates. A study by Giossi et al. has demonstrated similar results. They concluded that regardless of drug dosage (prophylactic/therapeutic), heparin is associated with better survival and reduced all-cause mortality [27]. Thus, based on the existing literature and our study findings, the administration of anticoagulative agents in critically-ill COVID-19 patients should be considered due to the high probability of thrombosis-related morbidities.

Another complication of the novel coronavirus infection, especially in critically-ill patients, is coinfection or super-imposed bacterial and fungal infections. In this study, 59 (81.9%) of acutely ill patients were prescribed antibiotics due to suspected or documented secondary infections. Our results showed an exceedingly higher rate of secondary bacterial infections compared to previous reports. A study by Clancy et al. [28] has reported that bacterial super-infections were observed in 13.5-44% of ICU-admitted patients. In a systematic review to determine the rate of bacterial super-/co-infection among patients with COVID-19, Langford et al. concluded that co-infections and super-infections were observed in 3.5% (95% CI: 0.4-6.7%) and 14.3% (95% CI: 9.6-18.9%), respectively [29]. We hypothesize that this discrepancy between our results and those in the current literature could be explained by the fact that all patients enrolled in this study were critically ill and in need of prolonged mechanical ventilation. Needless to say, there is an increased risk of secondary bacterial/fungal infection in mechanically-ventilated patients. Carbapenems, a last-resort antibiotic for Gram-negative bacteria, were previously proven to be an effective agent in the treatment of ventilator-associated pneumonia [30]. Similarly, our study demonstrated a significant association between the effects of carbapenems and survival. Similar results were observed regarding linezolid, an effective antibiotic for multi-drug-resistant Gram-positive organisms. Since antibiotics have no therapeutic effect on viral pneumonia and could lead to the increased selective pressure of antibiotic resistance, they should be reserved for COVID-19 patients with documented or suspected secondary infections.

This study has a few limitations. Primarily, it was a retrospective cross-sectional study and we were unable to compare the tracheostomy patients' variables with those of intubated (or other routes of mechanical ventilation) COVID-19 patients. Given the non-randomization of participants along with the fact that the patients enrolled for tracheostomy tube insertion had poor outcomes (compared with the general population), the potential for selection/Berkson’s bias should be taken into account. Also, the study had a relatively low sample size. Despite these limitations, this study involves one of the largest series evaluating characteristics and outcomes in patients receiving tracheostomy for COVID-19 infection. Another strength of this study is that we sought to determine the role of various factors including laboratory results and blood-gas analysis in patient outcomes, as well as the role of medications that are commonly used for patients with severe COVID-19 and their association with survival.

## Conclusions

The major indication for tracheostomy insertion for patients with severe COVID-19 infection is the need for prolonged mechanical ventilation. However, the mortality rate of these patients remains high despite receiving a tracheostomy. Our study concluded that percutaneous endoscopic tracheostomy seems to be the superior approach compared with an open tracheostomy. This study demonstrated that higher levels of HCO_3_, FiO_2_, and PaCO_2_ and a lower PaO_2_/FiO_2_ ratio are the main predictors of adverse outcomes. In addition, the administration of anticoagulative agents in critically ill COVID-19 patients should be taken into consideration due to the high probability of thrombosis-related morbidities.
